# Inventory Management of Health Commodities in a Tertiary Teaching Hospital in Ethiopia

**DOI:** 10.3389/fphar.2022.763909

**Published:** 2022-04-11

**Authors:** Nanati Legese, Dawit Teshome, Teferi Gedif

**Affiliations:** ^1^ School of Pharmacy, College of Health and Medical Sciences, Haramaya University, Harar, Ethiopia; ^2^ Department of Pharmaceutics and Social Pharmacy, School of Pharmacy, College of Health Sciences, Addis Ababa University, Addis Ababa, Ethiopia; ^3^ Department of Pharmaceutics and Social Pharmacy, School of Pharmacy, College of Health Sciences, Addis Ababa University, Addis Ababa, Ethiopia

**Keywords:** ABC analysis, ABC-VEN matrix, inventory management, medicines, laboratory commodities, medical supplies, Saint Paul Hospital Millennium Medical Collage, Ethiopia

## Abstract

**Background:** Analyzing purchased health commodities based on their budgetary consumption and importance is crucial for efficient utilization of a hospital’s budget. However, it is rarely seen when hospitals, mostly in developing countries, conduct such kinds of analyses and make an informed decision, including to utilize their limited budget efficiently. Therefore, the purpose of this study was to analyze a 3-year inventory of health commodities (medicines, medical supplies, and laboratory reagents and chemicals) in Saint Paul Hospital Millennium Medical College (SPHMMC).

**Methodology:** The study was conducted in SPHMMC located in Addis Ababa, Ethiopia. It is one of the largest specialized public hospitals in the country. It is a huge teaching hospital in the country where a large amount of budget is utilized. Three years of data were collected and ABC, VEN, and ABC-VEN matrix techniques were applied for the analysis. The data collection period was from March to April 2017.

**Results:** An average of 296 medicines, 194 laboratory commodities, and 105 medical supplies were purchased over 3 years. Class A medicines, which consume 80% of the total annual pharmaceutical expenditures (APE) account, are 17.8%–20% of the total medicines by quantity. Antibiotics (ceftriaxone 1 gm injection and metronidazole), IV fluids (sodium chloride 0.9% injection and dextrose 40% injection), and antidiabetic medication (insulin zin suspension and metformin) are among the top 10 medications by value that consume significant amounts of the budget of the hospital. On VEN analysis, an average of 24% of the items were vital, 67% were essential, and 4–8–8.9% were nonessential. Nonessential items consumed 0.49%, 9.9%, and 1.1% of Annual Expenditures (AEs) in 2013/14, 2014/15 and 2015/16, respectively. On ABC-VEN matrix analysis, a single expensive and nonessential medicine (valganciclovir HCL 450 mg tablet) consumed 9.4% of expenditure in 2014/15. Class A laboratory commodities, which consume 70%–80% of the total laboratory expenditures represented 8.5%–20% of the total laboratory commodities analyzed for the 3 years. From class A items, antimonoclonal antibodies in 2013/14, hemocue glucose 201 4 × 25 tests in 2014/15, and glucose tests in 2015/16 consumed the highest percentages: 9.2%, 8.2%, and 23.7% of the AEs, respectively. There were laboratory commodities procured out of the VEN list, and these accounted for 6.8%–31.2% of the total laboratory expenditures over the 3 years. Class A medical supplies, which consumed 80% of the total medical supply expenditures, represented only 8.2%–15.8% of the total items over the 3 years studied. Surgical gauze 90 cm × 100 m, surgical gloves sterile latex number 7.5, and examination gloves were the top three based on expenditures in all the studied years. In 2015/16, examination gloves alone consumed 71.9% of the total expenditure.

**Conclusion:** SPHMMC manages large numbers of health commodities (more than 500 excluding program commodities) which necessitate efficient inventory management practice in place. However, the purchase of the commodities particularly those products used for laboratory diagnosis is not strictly based on the hospital’s VEN list, indicating the need for better communication of the laboratory unit with the Drug and Therapeutic Committee (DTC) of the hospital. The DTC of the hospital should update the VEN list of the health commodities and strictly enforce the hospital procurement to adhere to the agreed upon list of medicines. In addition, the hospital should prioritize and decide the quantity and frequency of ordering health commodities based on regular ABC-VEN results.

## Introduction

At least half of the world’s population cannot access essential health services (World Health Organization, 2017). In many developing countries, including Ethiopia, patients experience a lack of essential medicines, leading to increased mortality and morbidity (World Health Organization, 2019). Besides this, it weakens health workers’ ability to treat their patients, and this, in turn, affects patients’ trust of a healthcare system (Mugisha et al., 2004; Hanson et al., 2005; Nabbuye-Sekandi et al., 2011).

Well-functioning inventory management helps health facilities use their scarce resources effectively and efficiently. There are several inventory control management methods, but the two commonly used are ABC analysis (classifying commodities based on budgetary consumption) and vital, essential, and nonessential (VEN) analysis. In ABC analysis, 10%–20% of items that consume 70%–80% of the budget are considered class A. The other 10%–20% items that consume 15%–20% and the remaining 60%–80% items that account for just 5%–10% of the budget are considered to be classes B and C, respectively. This classification is based on the Pareto principle (Quick et al., 1997; Holloway and Green, 2003; Management Sciences for Health and World Health Organization, 2007; WHO, 2012).

ABC analysis would let effective control of about two thirds of the total expenses by controlling only one fourth of the products (Kant et al., 1997). However, medicine expenditures are rarely analyzed and reported in most hospital pharmacy departments, especially in developing countries despite the inadequacy of the budgets (Lyombe, 2013).

Similarly, VEN analysis categorizes health commodities based on their criticality for the patients into three categories: vital, essential, and nonessential. Vital health commodities are given values based on their potential for lifesaving or being crucial for health services and prevention of death or disability of the patient. Essential health commodities are effective against less severe but significant illness. They are lifesaving, without which a patient may be in difficulty, but may be somehow substituted, and nonessential health commodities are effective for minor illnesses and have low therapeutic advantage (Management Science for Health, 2012).

A coupling of ABC and VEN analysis (ABC–VEN matrix) classifies health commodities into three categories, and this applies to substantial control of the commodities (Gupta et al., 2007). Category I comprises all V and A items (i.e., AV, BV, CV, AE, AN). Category II comprises the remaining items of the E and B groups (i.e., BE, CE, BN). Category III contains nonessential and inexpensive items (CN) (Devnani et al., 2010).

In this study, ABC–VEN analysis was carried out for the health commodities available in the pharmacy store of Saint Paul Hospital Millennium Medical College (2021), where no such analysis was conducted before. The result would serve as a baseline for proper management of the inventory in the hospital.

## Methodology

### Study Setting

SPHMMC, established in 1968, is the second largest specialized public hospital in Ethiopia. The hospital has more than 2,800 clinical and nonclinical staff that provide specialty services to patients who are referred from all over the country. It provides services for more than 700 inpatients and 1,200 emergency and outpatient clients daily (https://sphmmc.edu.et/). In addition to service, the hospital conducts various basic and applied research and serves as a training center for medicine and nursing students. It is a huge hospital in the country where a large amount of budget is utilized.

### Study Design and Period

This research is a retrospective, facility-based, cross-sectional study. ABC, VEN, and ABC-VEN matrix analysis techniques are used to assess the 3-year (2013/14 to 2015/16) health commodity consumption data of the hospital. Data were collected from March to April 2017.

### Inclusion and Exclusion Criteria

Health commodities (i.e., medicines; laboratory reagents, chemicals, supplies; and consumable medical supplies) purchased by the hospital through the Revolving Drug Fund (RDF) or donated to the hospital and recorded on good receiving vouchers (i.e., Model 19, Goods Receiving Notes, Delivery notes) in the years 2013/14, 2014/15, and 2015/16 are included. All program commodities, such as antiretroviral drugs, antituberculosis drugs, family planning drugs, and implantable equipment, were excluded from the study because they are vertically funded, managed, delivered, and monitored programs.

### Data Collection and Analysis

Data was collected by trained pharmacists from the hospital’s database system and documents (VEN list, delivery notes, and good receiving vouchers record (Model 19)). The quality of the data was confirmed by cross-checking documents containing the same information.

Annual consumption and expenses of each health commodity of the pharmacy was recorded in and analyzed using an MS Excel spreadsheet. First, ABC analysis was done by multiplying the unit cost of each item by its annual consumption. The resulting amounts were summed, the percentage was calculated for each item, and they were arranged in descending order of cost. Commodities were classified into A, B, and C categories according to cumulative cost disbursed. Then, VEN analysis was conducted based on the hospital’s VEN list. The proportions of the commodities in each category were computed together with their respective percentage expenditures. Finally, the ABC-VEN matrix was framed by cross-tabulating the ABC and VEN analysis that was used to get the different categories of health commodities, and the first and second alphabets represent the commodity’s place in the ABC and VEN analysis, respectively.

## Results

### Annual Expenditures on Health Commodities at SPHMMC

St. Paul Hospital purchased a total of 272–318 medicines, 170–223 laboratory commodities, and 97–118 medical supplies by type over the 3-year (2013/14, 2014/15, and 2015/16) period. In the first 2 years, the total annual expenditure on medicines was higher than the other health commodities (41.7% in 2013/14 and 54.2% in 2014/15). In 2015/16, the share of expenditure on medical supplies was much higher than the shares of the other health commodities (44.3%) (Table 1).

**TABLE 1:** Annual expenditures on health commodities at SPHMMC for 2013/14–2015/16.

	Medicine		Laboratory commodity		Medical supply		Total AE in ETB
	Quantity	AE in ETB (%)	Quantity	AE in ETB (%)	Quantity	AE in ETB (%)	
2013/14	272	18893252.8 (41.7)	170	8285439.3 (18.3)	97	18181634.2 (40.1)	45360326
2014/15	298	26662985.9 (54.2)	188	7001361.5 (14.2)	101	15544069.1 (31.6)	49208417
2015/16	318	21646483.0 (27.4)	223	22376833.3 (28.3)	118	35073204.7 (44.3)	79096521

AE, Annual Expenditure; ETB, Ethiopian Birr (1 US dollar was 21.4 ETB with 2016 average exchange rate).

### ABC-VEN Analysis of Medicines

**FIGURE 1:**
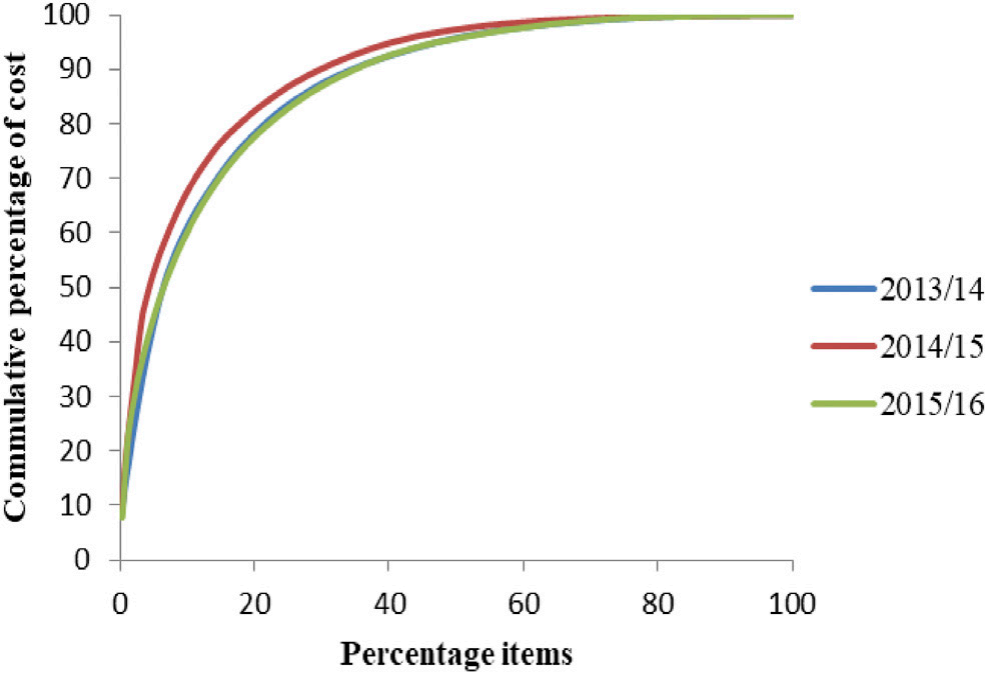
ABC analysis curve of medicines in SPHMMC (2013/14–2015/16), March 2017.

**FIGURE 2:**
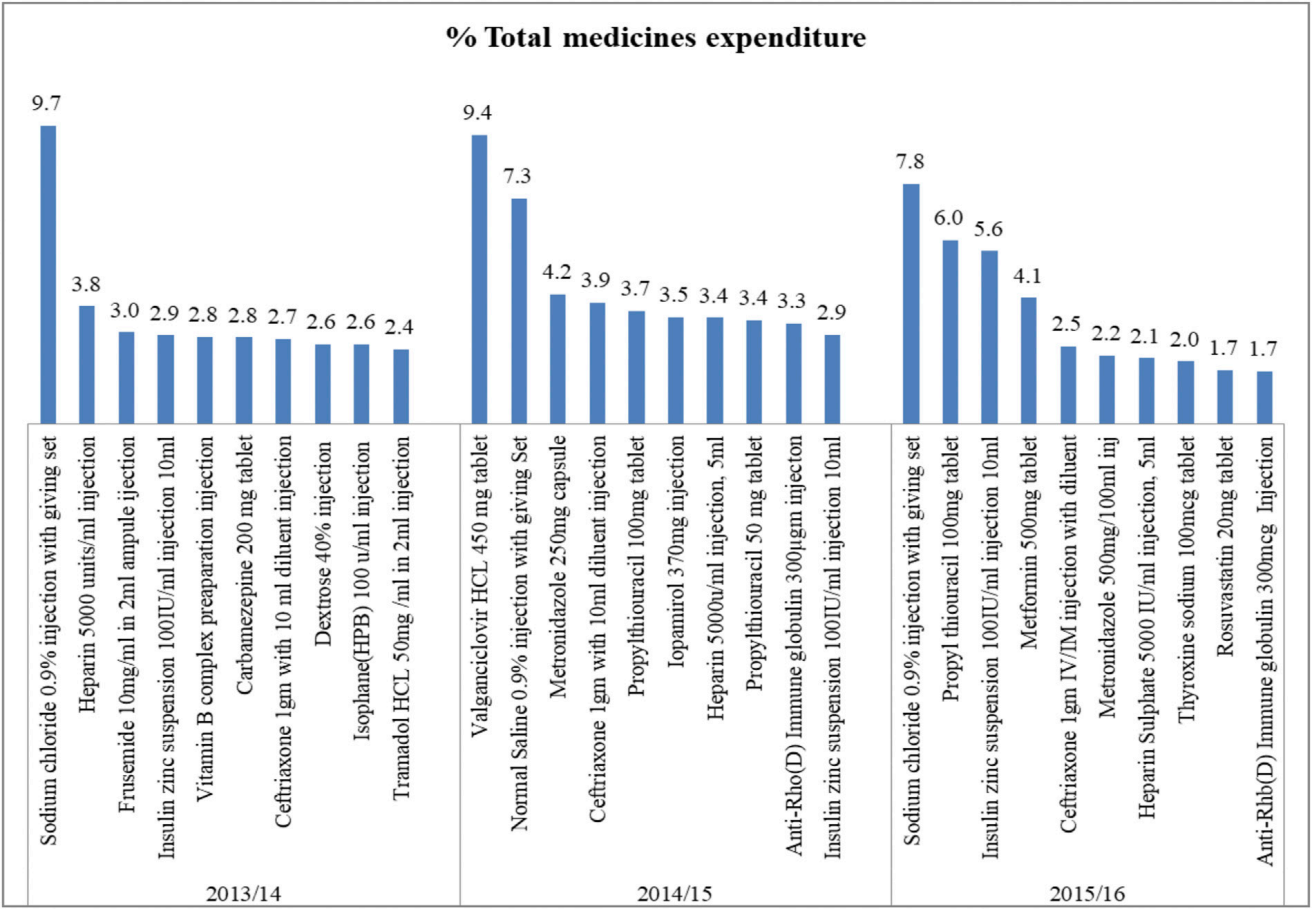
Top ten (based on cost) class “A” medicines in SPHMMC (2013/14–2015/16), March 2017.

A total of 272, 298, and 318 medicines were purchased in 2013/14, 2014/15, and 2015/16, respectively. Class A medicines, which consumed 80% of the total annual pharmaceutical expenditures (APE), account for 17.8%–20% of the total medicines by quantity (Figure 1). Antibiotics (ceftriaxone 1 gm injection and metronidazole), IV fluids (sodium chloride 0.9% injection and dextrose 40% injection), antidiabetic medication (insulin zin suspension and metformin), and anticoagulants (heparin), and immunoglobulin (Anti-Rh(D) immune globulin 300 mcg immune globulin injection) are the top 10 medications by value that consumed a significant part of the budget of the hospital. Figure 2 shows the details of these products.

The VEN analysis showed that V, E, and N items, on average, accounted for 24, 67, and 6% of the medicines procured by value over the 3-year period, respectively. The findings also reveal procurement of medicines (two to seven by quantity and 0.07%–0.36% by value) that were not included in the VEN list (Table 2).

**TABLE 2:** VEN analysis of medicines at SPHMMC for 2013/14–2015/16.

Year	Parameter	Medicines			Laboratory commodity			Medical supply		
		No. items	% Items	% Expenditure	No. Items	% Items	% Expenditure	No. Items	% Items	% Expenditure
2013/14	V	70	25.7	47.6	34	20.0	34.4	34	35.1	80.7
	E	187	68.8	51.8	79	46.5	42.6	54	55.7	14.98
	N	13	4.8	0.49	9	5.3	2.6	7	7.2	4.3
	NA	2	0.7	0.15	48	28.2	20.4	2	2.1	0.05
2014/15	V	73	24.5	45.1	35	18.6	38.9	36	35.6	74.2
	E	200	67.1	44.9	64	34.0	28.6	55	54.5	23.4
	N	18	6.0	9.9	1	0.5	1.3	7	6.9	2.34
	NA	7	2.3	0.07	88	46.8	31.2	3	3.0	0.07
2015/16	V	67	21.1	42.8	36	16.1	43.6	42	35.6	87.7
	E	218	68.6	55.7	105	47.1	48.9	62	52.5	10.4
	N	26	8.2	1.11	21	9.4	0.84	12	10.2	1.45
	NA	7	2.2	0.36	61	27.4	6.75	2	1.7	0.5

### ABC-VEN Matrix Analysis

Based on the analysis, 34%–39% of the items were found to be in category I (AV, AE, AN, CV, BV) accounting for more than 84%–86% of the annual expenditures of the hospital (Tables 3, 4). In 2014/15, a single AN medicine (valganciclovir HCL 450 mg tablet) consumed 9.4% of expenditure (Table 5). About 56%–60% of items were found to be in category II (BE, CE, BN), accounting for about 13%–15% of the annual expenditure over the years studied. Category III (CN) items accounted for 4%–7% of the medicines, accounting for less than 1% of expenditure (Table 3).

**TABLE 3:** ABC-VEN matrix analysis of medicines at SPHMMC for 2013/14–2015/16.

Year	Category	Medicines			Laboratory commodity			Medical supply		
		No. items	% Items	% Expenditure	No. Items	% Items	% Expenditure	No. Items	% Items	% Expenditure
	I	108	40.0	86.6	52	42.3	69.5	38	40.0	88.3
2013/14	II	150	55.6	13.16	62	50.4	9.4	53	55.8	11.5
	III	12	4.4	0.13	8	6.5	0.73	4	4.2	0.18
	I	101	34.7	85.5	54	54.0	61.1	42	42.9	85.9
2014/15	II	175	60.1	14.3	48	48.0	7.7	51	52.0	13.6
	III	15	5.2	0.16	—	—	—	5	5.1	0.36
	I	107	34.4	83.6	47	29.0	83.6	43	37.1	89.8
2015/16	II	180	57.9	15.5	96	59.3	9.3	62	53.4	9.4
	III	24	7.7	0.54	19	11.7	0.32	11	9.5	0.42

**TABLE 4:** Selected Vital and Expensive (AV) health Commodities in SPHMMC (2013/14-2015/16), March 2017.

	Item’s name
Medicines	Sodium chloride 0.9% injection (normal saline) with giving set iv infusion
	Insulin zinc suspension 100 IU/ml injection (NPH), lent 10 ml
	Ceftriaxone 1 gm IV/IM injection with diluent
	Metronidazole 500 mg/100 ml injection
	Heparin sulphate 5000 IU/ml injection, 5 ml
	Anti-Rhb(D) Immune Globulin 300 mcg Immune globulin Injection
	Iopamirol 370 mg injection
	Dextrose in Normal Saline 5%+0.9% in 1000 ml IV infusion injection
	Vancomycin 1 g injection
	Hydralazine 20 mg/ml injection
	Ringer’s injection solution each 1000 ml contains with giving set Iv infusion (injection)
	Tetanus antitoxin 1500 IU/ml in 1 ml injection
	Cimetidine 200 mg/ml in 2 ml ampule injection
	Salbutamol (albuterol) 0.1 mg/dose aerosol (oral inhalation)
	Ampicillin sodium 500 mg injection
Medical supplies	Examination glove sterile medium size
	Surgical glove no 7.5 pair
	Gauze surgical 90 cm × 100 m
	Syringe with needle of different sizes (3, 5, 10, 20 cc)
	Disposable syringe (Sterile) three parts, 10 ml Luer fitting with 21G 1 1/2 needle
	Catgut chromic gauge 5.0 (1) 75 cm on 48 mm ½ circle round bodied heavy needle
	Intravenous cannula set 24G
	Gauze bandage 12.5 cm × 5 m
	Intravenous cannula set 18G
	Disposable syringes (sterile) three parts, 5 ml Luer fitting with 21G needle
	Catgut chromic gauge 5.0 (2/0) 75 cm on 48 mm ½ circle round bodied heavy needle
Laboratory reagents, chemicals and supplies	Glucose test
	Serum separator tube (ss tube)
	Hemcue glucose RT 201 4 × 25
	EDTA test tube
	Troponin (roch-cobas E-411)
	Iodine 2% tincture 1000 ml
	Urographin (magluminet + Sodium diatrizoate) 76%
	Anti D monoclonal
	Fluid pack
	ALP (roch-cobas integra)
	UREA/BUN
	SGPT
	SGOT test

**TABLE 5:** AN subgroups of category I Health Commodities in SPHMMC (2013/14-2015/16), March 2017.

Year	Item name	Commodity type	% Expenditure
2013/14	TG GPO- PAP	Laboratory	1.88
	Surgical scalpel blade size number 15	Medical supply	1.98
	Adhesive plaster zinc oxide size 7.5 × 10 m	Medical supply	1.72
2014/15	Valganciclovir HCL 450 mg tablet	Medicine	9.40
	Neoplastin cl2	Laboratory	1.30
	Adhesive Plaster zinc oxide size 7.5 cm × 10 cm	Medical supply	1.68
2015/16	—	—	—

### ABC-VEN Analysis of Laboratory Reagents, Chemicals, and Supplies

**FIGURE 3:**
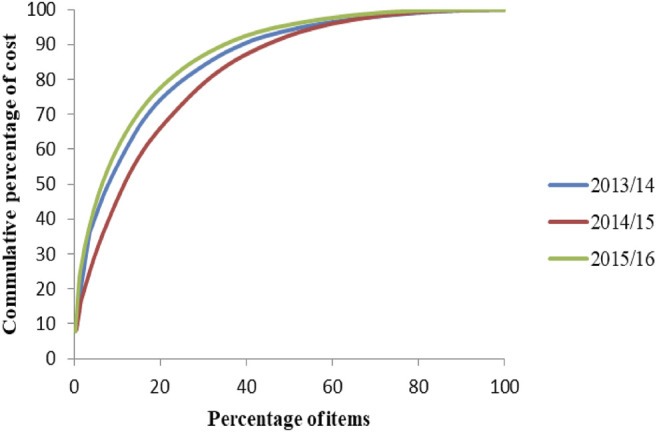
ABC analysis curve of laboratory commodities in SPHMMC (2013/14–2015/16), March 2017.

**FIGURE 4:**
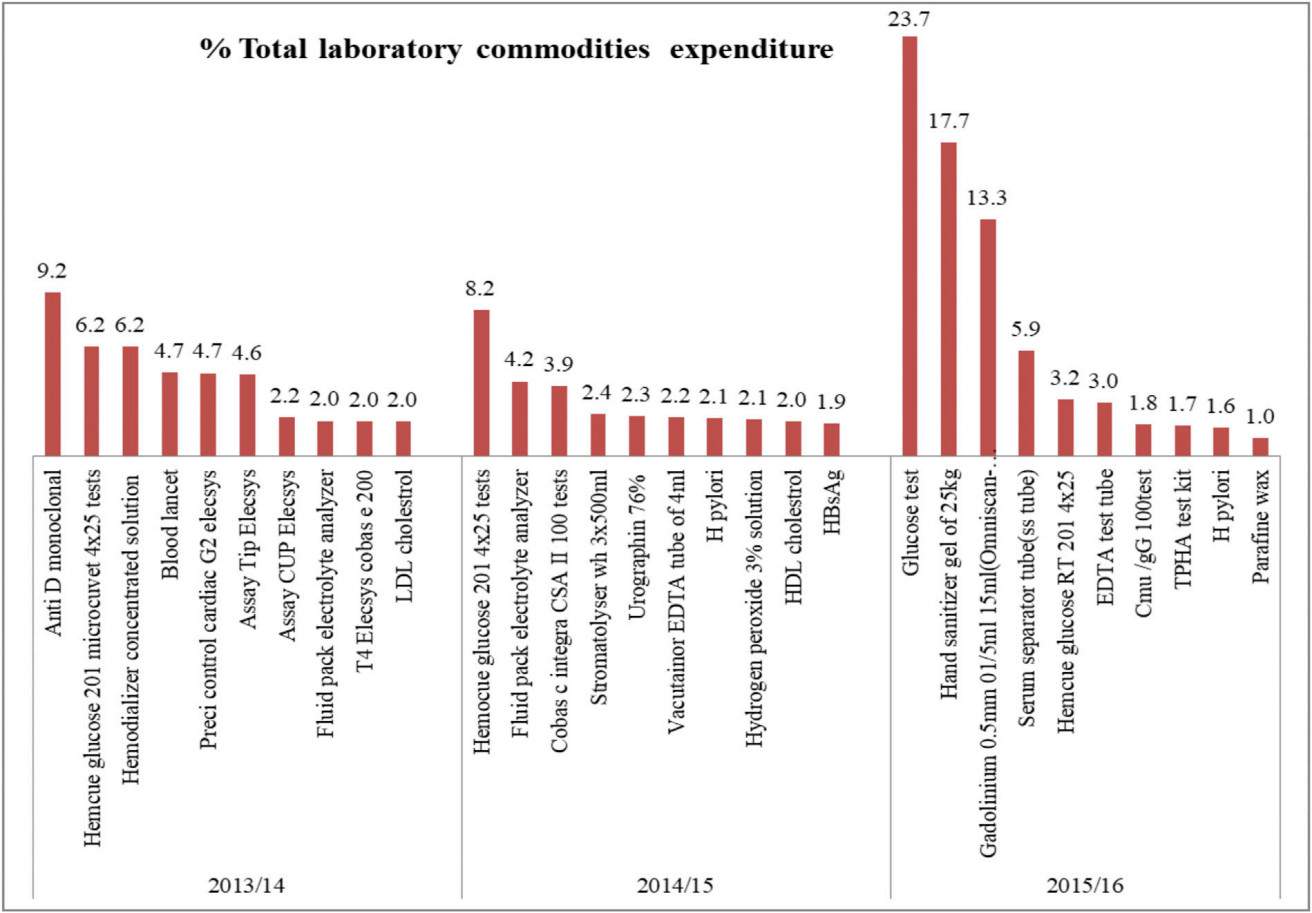
Top ten (based on cost) class “A” laboratory commodities in SPHMMC (2013/14–2015/16), March 2017.

A total of 170, 188, and 223 laboratory commodities (laboratory reagents, laboratory supplies and chemicals) were purchased in 2013/14, 2014/15, and 2015/16 respectively. Class A laboratory commodities represented 8.5%–20% of the total laboratory commodities analyzed for the 3 years (Figure 3). Antimonoclonal antibodies (9.2%), hemocue glucose 201 4 × 25 tests (6.2–8.2%), fluid pack electrolyte analyzer (4.2%), glucose tests (23.7%), hand sanitizer gel (17.7%), and gadolinium 0.5 mm 01/5 ml 15 ml MRI contrast agent (13.3%) were the items that consumed a significant part of the budget of the laboratory commodities (Figure 4).

The VEN analysis showed 16.7%–20.5% (35–37) of laboratory commodities were vital, 67% (188–219) were essential and the rest were nonessential. Unlike medicines, there were a number of laboratory commodities procured out of the VEN list. These accounted for 49 (28.8%), 89 (47.3%), and 62 (27.8%) items of the total procurement in 2013/14, 2014/15, and 2015/16, respectively. These items accounted for 6.8%–31.2% of the total laboratory expenditures over the 3 years (Table 2).

### ABC-VEN Matrix Analysis

Based on the analysis result, 21.5%–31% items were found to be category I, accounting for 61%–83.6% of laboratory expenditures over the 3 years (Table 3). Selected vital and expensive (AV) laboratory commodities over the 3 years are shown in Table 4. Expensive and nonessential (AN) items, TG GPO- PAP and neoplastin cl accounted for 1.9% and 1.3% of the total annual laboratory expenditures in 2013/14 and 2014/15, respectively (Table 5). About 25.5%–37% of items were found to be category II (BE, CE, BN), accounting for 7.7%–9.4% of annual laboratory expenditures in the years studied. Category III (CN) items accounted for 4.7% and 8.5% of the laboratory commodities, accounting for less than 1% of expenditures in 2013/14 and 2015/16 although there was no category III item in 2014/15 (Table 3). There were a number of laboratory commodities that were not classified in the three categories of the ABC-VEN matrix because they were not included in the VEN classification of the hospital. As can be seen from Table 6, significant cost was spent on these health commodities, especially on class A groups.

**TABLE 6:** Class A Laboratory commodities that were not included in the VEN classification of SPHMMC, March 2017.

Year	Item name	% Expenditure	Total % expenditure
2013/14	Assay Tip Elecsys	4.60	11.68
	Assay CUP Elecsys	2.19	
	T4 Elecsys cobas e 200	1.98	
	CA 15-3 G2 Elecsys cobas e 100	1.20	
	LH G2 CS elecsys	0.98	
	Cell check Elecsys	0.72	
2014/15	Cobas c integra CSA II 100 tests	3.95	15.1
	Stromatolyser wh 3 × 500 ml	2.40	
	TP urine + CSF of 150 tests	1.59	
	Ck prest 5(PTT)	1.36	
	Ck prest 2(PTT)	1.16	
	HBsAg quant G2 elesyscobas of 100	1.08	
	Uanc 200 tests cobas integra	1.05	
	Plussed tube	1.03	
	Cuvettes for hemoce	0.77	
	PT vacuum tube	0.72	
	Total expenditure	15.1	
2015/16	Cmu/gG 100 test	1.79	1.79

### ABC–VEN Analysis of Medical Supplies

**FIGURE 5:**
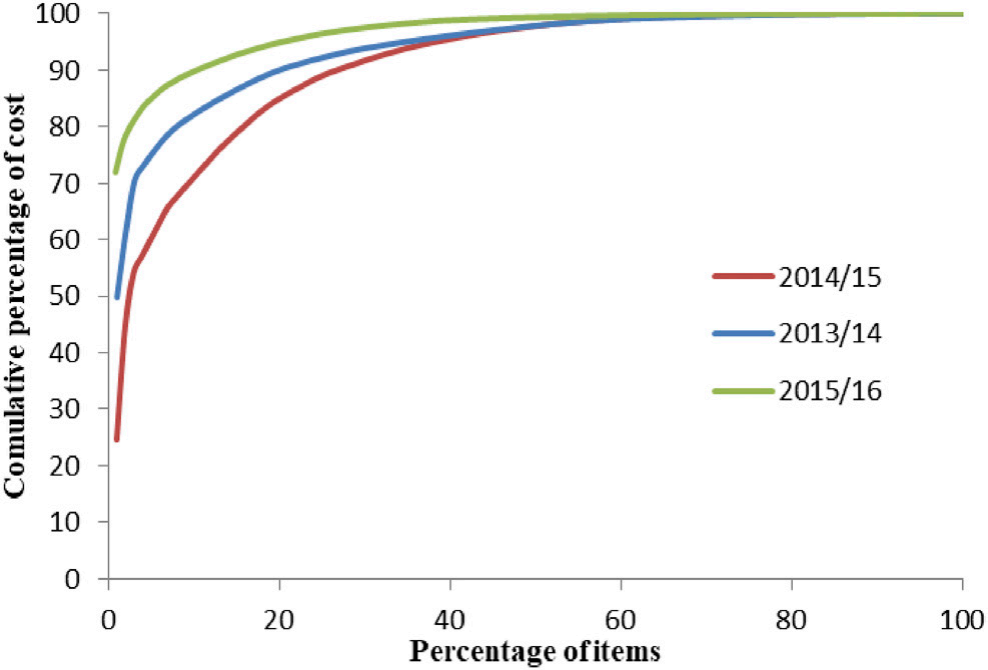
ABC analysis curve of medical supplies in SPHMMC (2013/14–2015/16), March 2017.

**FIGURE 6:**
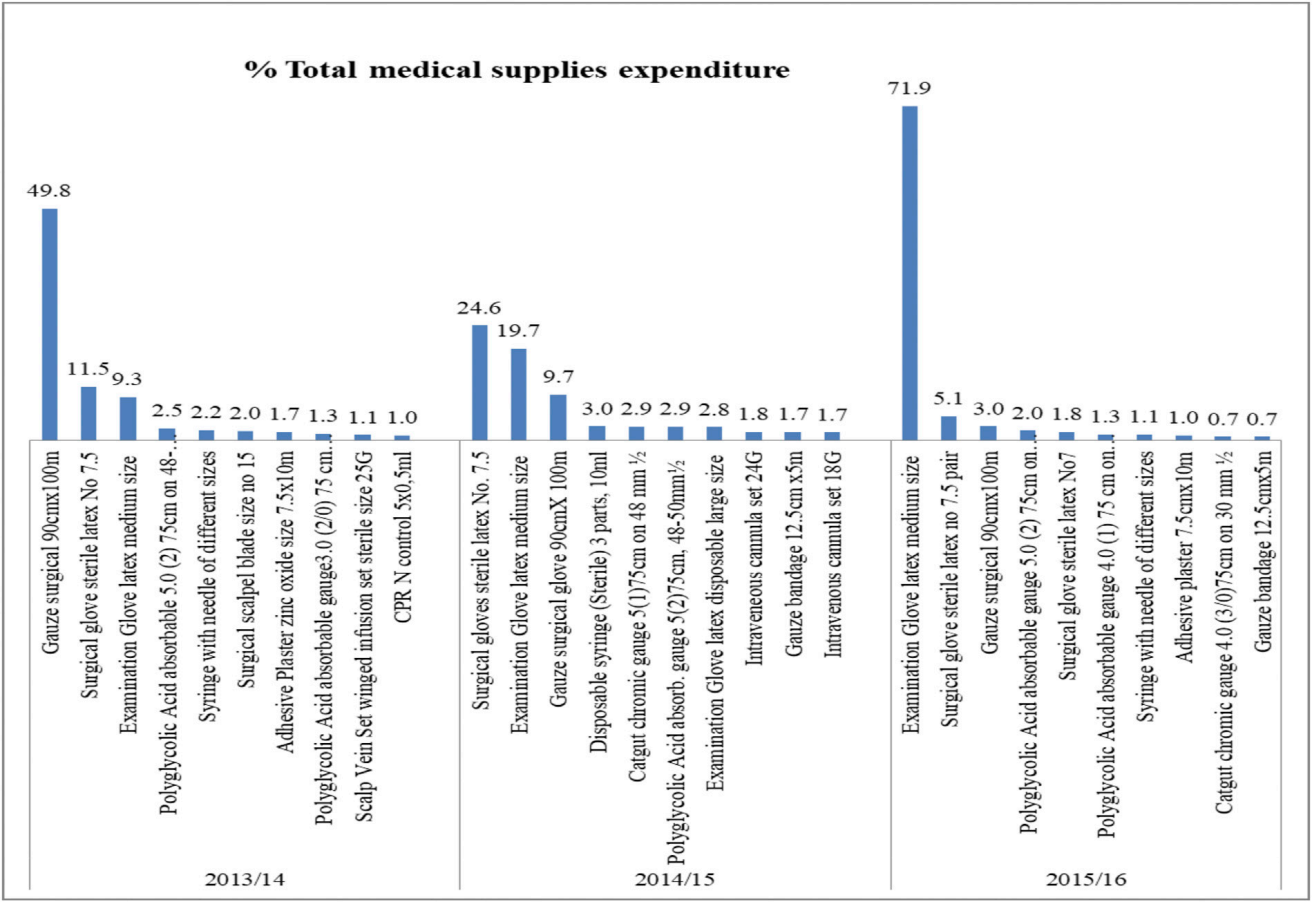
Top ten (based on cost) class “A” medical supplies in SPHMMC (2013/14–2015/16), March 2017.

A total of 97, 101, and 118 medical supplies were purchased in 2013/14, 2014/15, and 2015/16, respectively. Class A medical supplies, which consumed 80% of the total medical supply expenditures, represented only 8.2%–15.8% of the total items over the 3 years studied (Figure 5). Surgical gauze 90 cm × 100 m, surgical gloves sterile latex number 7.5, and examination gloves were the top three based on expenditures in all the studied years. In 2015/16, 80% of total expenditures were taken by 3 (2.5%) of the total medical supplies, and examination gloves alone consumed 71.9% of the total expenditure. The 10 top medical supplies based on expenditures are shown in Figure 6. Class C medical supplies represented the highest number of different medical supplies, consuming only an average of 5% of the total expenditure.

The VEN analysis showed that V accounted for an average of 35–43 (36%) of the different medical supply types procured over the 3 years, E for 55–63 (53%–56%) and N accounted for 7–13 (6.9%–10%) of different medical supply types procured. The findings also reveal that there were medical supplies procured out of the VEN list during the 3 years. These items accounted for 0.05, 0.07, and 0.43% annual expenditure in 2013/14, 2014/15, and 2015/16, respectively (Table 2).

### ABC–VEN Matrix Analysis

Based on the result of the analysis, 36%–41% items were found to be category I, accounting for more than 85.9%–88.3% of annual medical supply expenditures in the 3 years studied (Table 3). Selected vital and expensive (AV) medicines over the 3 years are shown in Table 4. Expensive and nonessential (AN) items, surgical scalpel blade size no. 15 (in 2013/14) and adhesive plaster zinc oxide size 7.5 × 10 m (in 2014/15 and 2015/16), consumed a huge amount of money (Table 5). About 50.4%–54% of items were found to be category II (BE, CE, BN), accounting for about 11.4%–13.7% of annual medical supply expenditures in the years studied. Category III (CN) items accounted for 4.2%–9.5% of the medical supply items, accounting for less than 1% of expenditures (Table 3).

### Morbidity Pattern Analysis

The annual morbidity for SPHMC is shown in Table 7. The morbidity data for 2013/14 were not available; consequently, this is not presented in Table 7. The annual morbidity data in descending order for 2014/15 and 2015/16 shows almost a similar pattern with trauma and other unspecified diseases of the digestive system having the highest number of cases.

**TABLE 7:** Morbidity pattern at SPHMMC for 2014/15-2015/16.

2014/15	
Diseases	*n* (%)
Trauma (injury, fracture etc.)	5913 (15.8)
Other or unspecified disease of digestive system	5698 (15.2)
Other or unspecified disease of circulatory system	4067 (10.8)
Other or unspecified disease of genitourinary system	3789 (10.1)
Other or unspecified disease of eye and adnexa	3692 (9.8)
Dental and gum diseases	3606 (9.6)
Disease of musculoskeletal system and connective tissue	2961 (7.9)
Neoplasms	2753 (7.3)
Hypertension and related diseases	2519 (6.7)
Diabetes mellitus	2516 (6.7)
**2015/16**	
**Diseases**	***n* (%)**
Trauma (injury, fracture etc.)	9350 (19.4)
Other or unspecified disease of digestive system	7249 (15.0)
Other or unspecified disease of genitourinary system	6279 (13.0)
Other or unspecified disease of eye and adnexa	5256 (10.9)
Neoplasms	3561 (7.4)
Other or unspecified disease of skin and subcutaneous tissue	3415 (7.1)
Dental and gum diseases	3404 (7.0)
Other or unspecified disease of circulatory system	3293 (6.8)
Hypertension and related diseases	3263 (6.7)
Diabetes mellitus	3189 (6.6)

## Discussion

As ABC analysis of the present study shows, class A medicines account for 17.8%–20% of the total purchased medicines (by quantity). Studies done in hospitals in different countries in Africa (Lyombe, 2013; Kokonya, 2016; Kivoto et al., 2018), Europe (GünerGören and Dağdeviren, 2017; Antonoglou et al., 2017), and Asia (Anil et al., 2012; Kumar and Chakravarty, 2015; Junita and Sari, 2012) report the same: class A items are few but very expensive requiring a close day-to-day control. Unless these items are managed properly, expenditures of hospitals will rise and affect the overall provision of healthcare services. Priority can be given for improving forecasting accuracy, shortening the time for ordering products, and determining the amount of holding safety stock and price discounts for very few but expensive items to manage the hospital’s budget efficiently (Management Science for Health, 2012).

Of the total medications consumed; sodium chloride 0.9% injection had the highest share of expenditure. Treating trauma (injury, fracture), which is the most prevalent condition in the hospital, might be the reason for the high consumption of this IV fluid. This finding is in line with a study conducted in Sudan in which sodium chloride solution 0.9% injection was ranked first by contributing 5% of total expenditures (Ahmed et al., 2019).

VEN classification of the present study also reveals that V, E, and N items account for 24%, 67%, and 4%–8% of the different medicines procured over the 3-year period, respectively. When comparing our study results with other studies, high variation was observed in the proportion of V, E, and N medicines. For instance, about 13.2%, 38.8%, and 48.0% of items were found to be V, E, and N items, respectively (Anand et al., 2013), and 32.41, 61.38, and 6.2% items were V, E, and N items, respectively in different hospitals in India (Khurana et al., 2013). These dissimilarities in the results could be because of differences in the availability of medicines (India is one of the largest producers of medicines) and specialty services and the way that classification and updating of medicines were done. The number of nonessential medicines has been increasing from year to year in SPHMMC (13 items in 2013/14 and 26 items in 2015/16). These items accounted for 0.5%, 9.9%, and 1.1% of annual expenditures over the 3 years, respectively. The current result is much different from a study in Tikur Anbessa Specialized University Hospital (TASUH), where there was no nonessential item purchased in the 3 years studied (Migbaru et al., 2016).

ABC–VEN matrix analysis in the present study shows that 34%–39% of items were found to be category I, accounting for more than 84%–86% of annual expenditures over the 3-year period. A similar study done in selected health facilities of the West Arsi Zone, Oromia, Ethiopia, revealed that category I items had the highest (84.7%) proportion of the total value of annual drug expenditure (ADE) (Jobira et al., 2021). Another study in Kenya shows that an average of 31% of medicine types in category I consumed an average of 85% of total drug expenditure (Kivoto et al., 2018). These medicines need exceptional inventory control and management. A single AN item (Valganciclovir HCL 450 mg tablet) in the present study consumed 9.4% of the annual expenditure in 2014/15. In a similar study conducted at Mwananyamala hospital in Tanzania, four of 233 medicines, which accounts for 7% of the total medicine expenditures, were expensive and nonessential (Mori et al., 2021). This subgroup (expensive and nonessential) needs removal from the list if possible or replacement with equivalent but less costly medicine; hence, it would bring cost savings without affecting patient care (Kokonya, 2016). Category II items (55.7%–60.1% in the 3 years) in the present study represented 13%–15% of the annual expenditure. These items can be ordered with a long interval and in bulk to gain from a reduced ordering cost. Category III items (4.4%–7.7%) took away only 0.13%–0.54% of the annual expenditure over the 3 years. These items can also be procured with a much longer frequency and in bulk to save ordering costs. Even though there are different pharmaceutical types and budgets in each health setting, for better control of inventories, narrowing pharmaceuticals down based on their cost and vitality is insisted on by different studies in different settings (Kumar and Chakravarty, 2015; Kokonya, 2016; Anand et al., 2013; Kritchanchai and Meesamut, 2015).

Among class A laboratory commodities, tests for glucose level represented a significant expenditure over the studied years. According to the 10 top diseases seen in SPHMMC, diabetes mellitus was the 10th with a prevalence of 6.6%–6.7%, which could be the reason for the higher expenditures on diabetic-related health commodities. On the ABC–VEN matrix analysis of laboratory commodities, expensive and nonessential items TG GPO- PAP and neoplastin cl accounted for 1.9% and 1.3% of the total expenditures in 2013/14 and 2014/15, respectively. It is recommended to remove these items from the list if possible or replace them with equivalent but less costly products. On the other hand, about 11.7 and 15.1% of the expenditure in the first 2 years studied was taken by class A laboratory commodities that were not on the essential drug list. These items should be reviewed by the drug and therapeutic committee (DTC) of the hospital for consideration of adding these items to the hospital’s VEN list if necessary.

Class A medical supplies, which consumed 80% of the total medical supply expenditures, represented only 8.2%–15.8% of the total items in 2013/14 and 2014/15. In similar study at a university hospital in Turkey, 12% of medical materials, accounting for 70% of annual medical materials expenditure, were categorized as class A (YĠĞĠ, 2017). In the current study, 80% of total medical supply expenditures were taken by 3 (2.5%) items only, from which examination gloves alone consumed 71.9% of the total supply expenditures in 2015/16. The study in Turkey found that the highest expenditure was on coronary stents, which accounted for less than 3% of the supply expenditure (YĠĞĠ, 2017). The expenditure on a single item in SPHMMC is very high, suggesting that strict control on its use would be gainful in saving costs. Surgical scalpel blade size no. 15 and adhesive plaster zinc oxide size 7.5 × 10 m were expensive and nonessential items identified in the present study. Revision and/or removal of these items from the list are indicated.

The limitation of this study is that the analysis used retrospective data; however, the findings are still relevant to date because, in developing countries, there are only a handful of published research papers that show the actual consumption of medicines, medical supplies, and laboratory reagents in a teaching hospital. Besides this, our findings are less likely to change over time because the pattern of diseases that frequently occur in teaching are more or less the same, limited generic medicines are supplied by a public supplier, and we included at least 3 years of data, which indicates a trend.

## Conclusion

SPHMMC manages large numbers of health commodities (more than 500, excluding program commodities), which necessitates efficient inventory management practice in place. However, the purchase of the commodities, particularly those products used for laboratory diagnosis are not strictly based on the hospital’s VEN list, indicating the need for better communication of the laboratory unit with the DTC of the hospital. The DTC of the hospital should update the VEN list of the health commodities and strictly enforce hospital procurement to adhere to the agreed upon list of medicines. In addition, the hospital should prioritize and decide the quantity and frequency of ordering health commodities based on regular ABC–VEN results.

## Data Availability

The raw data supporting the conclusions of this article will be made available by the authors, without undue reservation.

## Abbreviations

AE, Annual Expenditures; DTC, Drug and Therapeutic Committee; ETB, Ethiopian Birr; MSH, Management Sciences for Health; SPHMMC, Saint Paul Hospital Millennium Medical College; TASUH, Tikur Anbesa Specialized University Hospital; VEN, Vital, Essential, Non-essential.
